# Metabolic-immunoregulatory subtypes reveal prognostic and therapeutic insights in multiple primary lung cancer

**DOI:** 10.3389/fimmu.2026.1827965

**Published:** 2026-07-15

**Authors:** Yuli Zhao, Hanyu Zhao, Hui Wu, Huimin Tao, Chunmin Yang

**Affiliations:** 1 School of Food & Pharmaceutical Science and Technology, Guangzhou College of Technology and Business, Guangzhou, China; 2 Department of Medical Oncology, The First Affiliated Hospital of Henan University of Science and Technology, Luoyang, China

**Keywords:** epithelial heterogeneity, immune microenvironment, metabolic reprogramming, multiple primary lung cancer (MPLC), single cell RNA sequencing

## Abstract

**Background:**

Multiple primary lung cancer (MPLC) is an increasingly recognized subtype characterized by distinct lesions with independent origins. While recent studies have profiled the immune landscape of MPLC, its tumor-intrinsic metabolic features and immunoregulatory interactions remain largely unexplored.

**Methods:**

Single-cell RNA sequencing data from 11 single primary lung cancer (SPLC) tumors and 8 samples from 4 MPLC patients were analyzed using dimensionality reduction, clustering, and cell type annotation. Subtype-specific metabolic features and intercellular communication patterns were investigated through pathway enrichment and cell-cell interaction analyses. A prognostic model was constructed using Lasso-Cox regression. Immune microenvironment characteristics were assessed using deconvolution algorithms and immune-related signatures. Drug sensitivity prediction and functional assays were performed to explore potential therapeutic implications.

**Results:**

This study identified a metabolically distinct malignant epithelial subpopulation enriched in MPLC tumors, characterized by upregulation of amino acid metabolism pathways and active MHC-II-mediated interactions with immunosuppressive CD4^+^ Treg cells and mast cells. A metabolism-based eight-gene prognostic model was developed and validated in independent lung adenocarcinoma cohorts, effectively stratifying patient survival outcomes. High-risk patients exhibited immunosuppressive tumor microenvironment features, reduced immunotherapy response potential, and distinct drug sensitivity profiles. Functional assays confirmed that key metabolic genes, spermine oxidase (SMOX) and spermine synthase (SMS), promoted tumor proliferation and invasion, accompanied by transcriptional changes in PI3K/mTOR pathway components, highlighting their potential roles in poor prognosis and therapeutic vulnerability.

**Conclusion:**

This study provides a systematic characterization of malignant subpopulations in MPLC, highlighting metabolic reprogramming and immunoregulatory features that contribute to poor prognosis. These findings provide a rationale for metabolism-based prognostic stratification and highlight potential therapeutic strategies to improve clinical outcomes.

## 1 Introduction

Lung cancer remains the leading cause of cancer-related morbidity and mortality worldwide, with non-small cell lung cancer (NSCLC) accounting for approximately 85% of all cases. In recent years, the incidence of multiple primary lung cancer (MPLC) has shown a marked increase due to the widespread use of low-dose computed tomography (LDCT) screenings (1, 2). MPLC is characterized by the synchronous or metachronous development of two or more primary tumors in the lungs, each arising independently and typically presenting distinct histopathological or molecular features (3). Compared to single primary lung cancer (SPLC), MPLC exhibits greater genetic and clonal diversity along with heterogeneous therapeutic responses, posing significant challenges for accurate diagnosis, prognosis assessment, and personalized treatment selection (4). Understanding the tumor-intrinsic features and immune microenvironmental landscapes of MPLC is therefore critical to improving clinical management.

Recent advances in single-cell transcriptomics have enabled high-resolution profiling of the tumor microenvironment (TME) of MPLC. Emerging evidence suggests that MPLC tends to exhibit an immunologically “cold” phenotype. For instance, “super-MPLC” cases show significantly reduced CD8^+^ T cell infiltration and lower PD-L1 expression compared to conventional NSCLC, reflecting a less inflamed immune milieu (5). Moreover, certain immunosuppressive cell populations appear specifically enriched in MPLC tumors. One such example is the F13A1^+^ M2-like macrophage subpopulation, which is significantly more abundant in MPLC than in SPLC. These cells communicate with tumor and stromal cells via immunosuppressive ligands such as SPP1 and CCL13, thereby shaping an immunosuppressive microenvironment and promoting immune evasion and tumor progression (6). In addition, MPLC tumors contain higher proportions of CD1C^+^ conventional dendritic cells type 2 (cDC2), which exhibit a more pro-inflammatory transcriptional profile relative to those in SPLC. A novel epithelial subpopulation has also been identified in MPLC, characterized by CLDN2^+^ alveolar type II (AT2)-like cells. These cells appear to reside in a relatively quiescent differentiation state but occupy a central position in the cell-cell communication network, contributing to the structural organization of the tumor tissue (7). Notably, AT2-like tumor cells have been implicated in suppressing anti-tumor immunity through interactions with B cells within tertiary lymphoid structures (TLSs), particularly in lesions with poor immune responses (8). Furthermore, tumor-infiltrating lymphocytes in MPLC include a distinct population of T cells expressing high levels of GITR (TNFRSF18), a co-stimulatory molecule of the TNF receptor superfamily commonly found on activated effector and regulatory T cells. This finding suggests that GITR may serve as a promising immunotherapeutic target specific to MPLC (7).

Metabolic reprogramming has emerged as a critical mechanism driving immune suppression in the tumor microenvironment (9). On the one hand, tumor cells consume excessive glucose, glutamine, and other nutrients, starving anti-tumor lymphocytes of the fuel needed for effective function (10). On the other hand, tumor-derived metabolites such as lactate, adenosine, and kynurenine directly suppress immune cell activity or induce immunosuppressive phenotypes, including regulatory T cells (Tregs) and myeloid-derived suppressor cells (MDSCs) (11, 12). In parallel, immune cells within the tumor microenvironment undergo metabolic adaptation: cytotoxic T cells and NK cells become functionally exhausted in nutrient-depleted, hypoxic conditions, while tumor-associated macrophages shift toward anti-inflammatory states by utilizing metabolites like lactate (13). These metabolic-immune interactions are increasingly recognized as key drivers of immune evasion and resistance to immune checkpoint blockade (10, 11). Targeting tumor metabolism, by restoring nutrient availability or neutralizing immunosuppressive by products has shown promising results in enhancing immunotherapy efficacy in preclinical studies (12, 14). Overall, deciphering the interplay between tumor metabolism and immunity is crucial for uncovering new therapeutic targets and improving cancer immunotherapy outcomes.

In summary, although immune differences between MPLC and SPLC have been previously reported, most existing studies have focused on the overall composition and functional states of immune cells. A systematic characterization of malignant epithelial states enriched in MPLC is still lacking, particularly regarding their roles in metabolic reprogramming and immunoregulatory modulation. In this study, we analyzed single-cell transcriptomic data from both MPLC and SPLC to investigate the heterogeneity of malignant epithelial subpopulations. We specifically aimed to delineate the metabolic features and immunoregulatory interactions of malignant epithelial cells enriched in MPLC. To bridge single-cell findings with clinical applications, we constructed a lung adenocarcinoma (LUAD) prognostic model based on the metabolic signatures of the MPLC-enriched malignant subpopulation using bulk RNA-seq data. We further characterized immune microenvironment differences and predicted immunotherapy response between risk groups. Finally, we identified potential therapeutic agents and experimentally validated the functional role of key metabolic genes. Through this integrative analysis, we sought to offer new insights into the pathogenesis of MPLC and to identify potential targets for therapeutic intervention.

## 2 Materials and methods

### 2.1 Collection of single-cell RNA sequencing datasets

We performed comprehensive integration of publicly available single-cell RNA sequencing data obtained from the GEO database, specifically incorporating datasets GSE131907 (15) and GSE200972 (7). The merged collection included 11 SPLC tumor samples (each from an individual patient) from GSE131907 and 8 MPLC tumor samples (two lesions per patient) from 4 MPLC patients in GSE200972. To minimize histology-related confounding, squamous cell carcinoma (SCC)-derived MPLC samples (MPLC_1 and MPLC_2) were excluded from downstream adenocarcinoma-focused epithelial analyses. To address potential technical variability between these distinct datasets, we implemented Harmony (16) algorithm batch effect normalization before proceeding for further biological analysis.

### 2.2 Single cell analysis and cell type annotation

A detailed examination of the single-cell expression matrix was conducted utilizing Scanpy (v1.9.1) (17). Cells and genes were filtered based on the following criteria: genes expressed in at least 3 cells (min_cells ≥ 3), cells containing at least 200 genes (min_genes ≥ 200), total counts per cell less than 3000 (genes_by_counts < 3000), and mitochondrial percentage below 20% (pct_counts_mt < 20). Principal component analysis (PCA) was first applied for dimension reduction, where the parameter “ov.pp.pca” was configured to “n_pcs=50” to preserve dominant variance patterns. Following PCA transformation and normalization, cell proximity relationships were determined with “n_neighbors=15, “ forming a connectivity graph for subsequent clustering. Technical batch effects were mitigated through Harmony integration (default parameters: theta= 2, lambda= 1, sigma= 0.1, max_iter= 10, early_stop= TRUE), enhancing data comparability. To further evaluate integration performance, quantitative batch-mixing metrics, including dataset-level (iLISI), cell-type-level (cLISI) local inverse simpson’s index (16), and silhouette scores were calculated before and after Harmony correction. The Leiden clustering method was executed at a resolution of 0.4 to balance cluster specificity. UMAP projections, generated via sc.pl.umap, provided a representation of cellular distributions. Cluster identities were annotated by combining CellTypist (v1.7.1) predictions with established marker genes, enabling biological interpretation (18, 19). Malignant cells were identified using inferCNV (cutoff = 0.1, HMM-based mode, denoise = TRUE) with normal lymphocytes as references (20). Epithelial cells exhibiting CNV scores exceeding the 95th percentile of reference cells were defined as malignant.

### 2.3 Differential expressed gene analysis

To detect genes with varying expression levels among clusters, the Wilcoxon rank-sum test was applied using sc.tl.rank_genes_groups. Transcripts were deemed significantly upregulated if their adjusted P-value was below 0.05 and their log_2_ fold change (FC) ≥ 2, while those with a log_2_ FC ≤ -2 were classified as downregulated.

### 2.4 Functional enrichment analysis

Gene Ontology (GO) and pathway enrichment were assessed using clusterProfiler (v3.18.1) with the org.Hs.eg.db reference database. Gene symbols were converted to Entrez IDs via the bitr function prior to analysis. Enrichment was evaluated for three GO domains: Biological Processes (BP), Molecular Functions (MF), and Cellular Components (CC), applying the enrichGO method with parameters: OrgDb = org.Hs.eg.db, ont = “BP/CC/MF”, pvalueCutoff = 0.05, and readable = TRUE. Statistically significant terms were filtered via the Benjamini-Hochberg correction (FDR < 0.05) and plotted as dot/bar graphs. For KEGG pathway analysis, the g:Profiler Python API was employed, querying the hsapiens genome annotation. DEG lists were analyzed with FDR-adjusted significance (adjusted P-value < 0.05), and results were visualized to highlight pathway associations. In addition, Gene Set Enrichment Analysis (GSEA) leveraged MSigDB hallmark gene sets to detect pathway-level alterations (FDR < 0.05). Gene Set Variation Analysis (GSVA, v1.38.1) computed ssGSEA scores to compare pathway activities between risk groups.

### 2.5 Cell-cell interaction analysis of cell clusters

To investigate the intercellular communication among CD4_Tn-LEF1, CD4_TM_NR4A1, CD4_Tfh-CXCL13, CD4_Treg-FOXP3, CD8_Tc-GZMK, Mast cell, and Malignant_CAPS cell clusters, CellChat (v1.4.0) was utilized (21). Normalized data and labels were processed using the CellChatDB.human database. Low-quality cells (<50) and weakly expressed genes were excluded. Overexpressed ligands/receptors were detected via identifyOverExpressedGenes, and interaction probabilities were computed with computeCommunProb. Pathway-level networks were aggregated via aggregateNet, and circle/heatmap plots highlighted key senders/receivers (P < 0.05).

### 2.6 Development and assessment of the prognostic model

To identify genes linked to overall survival (OS) in TCGA-LUAD cohorts, univariate Cox regression was performed (P < 0.05). Candidate prognostic genes were subsequently subjected to least absolute shrinkage and selection operator (LASSO)-penalized Cox regression using the “glmnet” R package (v4.1-2) (22, 23). A 10-fold cross-validation procedure was applied to determine the optimal penalty parameter (lambda) and minimize overfitting. The optimal lambda value was selected based on the minimum cross-validation error (lambda.min=0.0118). A risk-scoring system was derived by weighting mRNA expression levels of selected genes by their regression coefficients. Eight genes were prioritized, and patients were stratified into high/low-risk subgroups using median risk scores. The predictive performance of the model was evaluated using Kaplan-Meier analysis (survival, v3.7-0/survminer v0.0.6 R packages) and externally validated on the GSE31210 and GSE30219 datasets.

### 2.7 Protein-protein interaction network analysis

To explore potential functional associations among the model genes, we constructed a protein-protein interaction (PPI) network using the STRING database (https://string-db.org) (24). The analysis was performed with default parameters, which include a minimum required interaction score of 0.4 (medium confidence) and the inclusion of both direct (physical) and indirect (functional) associations. Only interactions supported by experimental data, curated databases, co-expression, or text mining were considered. The resulting network was visualized using STRING’s embedded viewer.

### 2.8 Tumor microenvironment analysis

Tumor immune and stromal content, as well as tumor purity, were inferred using the ESTIMATE (v1.0.13) algorithm (25). The Tumor Immune Dysfunction and Exclusion (TIDE) algorithm (26) was used to calculate TIDE scores, which predict potential responsiveness to immune checkpoint blockade therapy. Immune cell composition was deconvoluted using both CIBERSORT (v0.1.0) (27) and EPIC (28) based on bulk transcriptomic profiles. Drug sensitivity was predicted using the pRRophetic (v0.5) package (29), integrating gene expression profiles from the PANCANCER_IC and CGP2016ExprRma datasets. T-cell receptor (TCR) repertoire analysis was performed to assess clonal expansion and diversity using metrics such as clonality and Shannon entropy derived from TCR sequencing data (30).

### 2.9 Cell culture

Human lung cancer cell lines A549 and H1299, as well as the normal bronchial epithelial cell line BEAS-2B, were obtained from CTCC: SCSP-503, CTCC: SCSP-589, CTCC: SCSP-5067, respectively. All cells were cultured in DMEM medium (Gibco) supplemented with 10% fetal bovine serum (FBS) (Gibco), 100 U/mL penicillin, and 100 μg/mL streptomycin, and maintained at 37 °C in a humidified atmosphere containing 5% CO_2_.

### 2.10 RNA isolation and quantitative PCR

Total RNA was extracted using TRIzol reagent (Invitrogen) according to the manufacturer’s protocol. cDNA was synthesized from 1 μg of total RNA using the PrimeScript RT Reagent Kit (Takara). Quantitative PCR was performed using TB Green Premix Ex Taq II (Takara) on a LightCycler 480 System (Roche). Gene expression was normalized to β-Actin, and relative expression was calculated using the 2^−ΔΔCt method. Primer sequences are listed in **Supplementary Table 2**.

### 2.11 Cell proliferation assay

Cell proliferation was assessed using the Cell Counting Kit-8 (CCK-8) (Dojindo). Briefly, cells transfected with siRNAs targeting spermine oxidase (SMOX) or spermine synthase (SMS), or a negative control siRNA (siNC), were seeded into 96-well plates at a density of 2, 000 cells per well. At 0, 24-, 48-, 72- and 96-hours post-seeding, 10μL of CCK-8 reagent was added to each well and incubated for 2 hours. Absorbance was measured at 450 nm using a microplate reader (Thermol). Each experiment was performed in triplicate.

### 2.12 Transwell migration assay

To test the effect of SMOX or SMS on lung cancer cell metastasis, cell invasion assays were performed using a BD BioCoat Matrigel Invasion Chamber (8 μm pore size) (BD Biosciences, USA). 1 × 105 cells were suspended in serum-free medium and added to the upper face of the cell culture chambers. The chambers were placed into a 24-well plate containing DMEM supplemented with 10% FBS. After incubation for indicated times (20 h for A549 and 24 h for NCI-H1299 cells, respectively), cells in the upper face of the chambers were scraped, and cells adhering on the lower surface of the chambers were fixed, stained with crystal violet, and photographed with a microscope (ZEISS Axio Observer Z1; Zeiss, Jena, Germany).

### 2.13 Statistics analysis

Group differences were tested via two-tailed t-tests (R/GraphPad Prism). Multivariate Cox models (survival package) identified OS-associated factors (FDR-adjusted P < 0.05). Data represent mean ± SEM from ≥ 3 replicates. The Wilcoxon rank-sum test (R implementation) determined significance, with FDR correction for multiple testing. The significance thresholds were defined as follows: *P < 0.05; **P < 0.01; ***P < 0.001.

## 3 Results

### 3.1 scRNA-seq atlas of MPLC and SPLC

**Figure 1:**
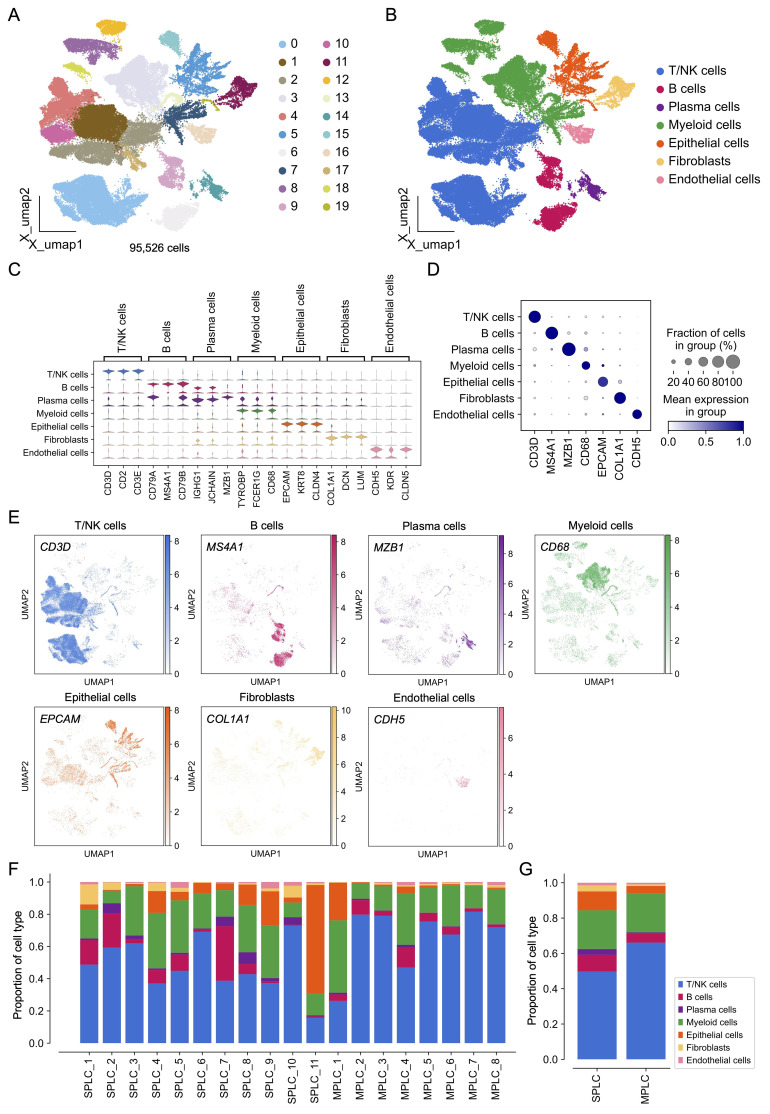
Single-cell transcriptomic analysis reveals cellular heterogeneity in MPLC and SPLC tumors. **(A)** UMAP plot of 95, 526 high-quality cells from 11 SPLC patients and 4 MPLC patients, colored by 20 unsupervised clusters. **(B)** UMAP plot annotated with seven major cell types identified through canonical marker gene expression. **(C)** Violin plots highlighting the cell type-specific expression of selected marker genes. **(D)** Dot plot displaying the expression of canonical marker genes for each cell type. **(E)** UMAP visualization of marker gene expression distribution across all cells for each major cell type. **(F)** Stacked bar plot showing the proportion of each cell type per individual sample. **(G)** Comparative stacked bar plot of cell type proportions between SPLC and MPLC groups.

To characterize the cellular composition and heterogeneity of multiple primary lung cancer (MPLC) and single primary lung cancer (SPLC), we collected and analyzed single-cell RNA sequencing (scRNA-seq) data of tumor samples from 11 SPLC patients with lung adenocarcinoma and 4 MPLC patients, each MPLC patient contributed two tumor samples. Available clinical information, including age, sex, histology, and smoking status, is summarized in **Supplementary Table 1**. After stringent quality control, a total of 95, 526 high-quality cells were retained for downstream analysis. Using the Uniform Manifold Approximation and Projection (UMAP) algorithm, these cells were projected into 20 distinct clusters (**Figure 1A**), suggesting that major cell-type structures were not dominated by dataset-specific effects (**Supplementary Figure 1A**). This was further supported by quantitative integration diagnostics, including iLISI and silhouette-based analyses, which showed increased dataset mixing (higher iLISI), reduced dataset-driven separation (lower silhouette), and preserved cell-type structure (**Supplementary Figure 1B**). Based on automated annotation and canonical marker gene expression, we identified seven major cell types (**Figure 1B**), including 51, 264 T/NK cells, 19, 276 myeloid cells, 6, 232 B cells, 6, 151 epithelial cells, 1, 005 endothelial cells, and 1, 528 plasma cells. The expression of representative marker genes for each cell type is shown in **Figure 1C**, demonstrating cell type-specific enrichment patterns (**Figures 1D, E**; **Supplementary Figures 1C–E**). We further visualized the proportion of each cell type across individual patients and observed a relatively balanced distribution among samples (**Figure 1F**). Notably, MPLC samples exhibited a higher proportion of T/NK cells and lower proportions of B cells and epithelial cells compared to SPLC. This lower epithelial cell proportion in MPLC is consistent with the typically smaller lesion sizes observed in MPLC patients (**Figure 1G**).

### 3.2 Malignant epithelial cell composition and metabolic features in MPLC and SPLC

**Figure 2:**
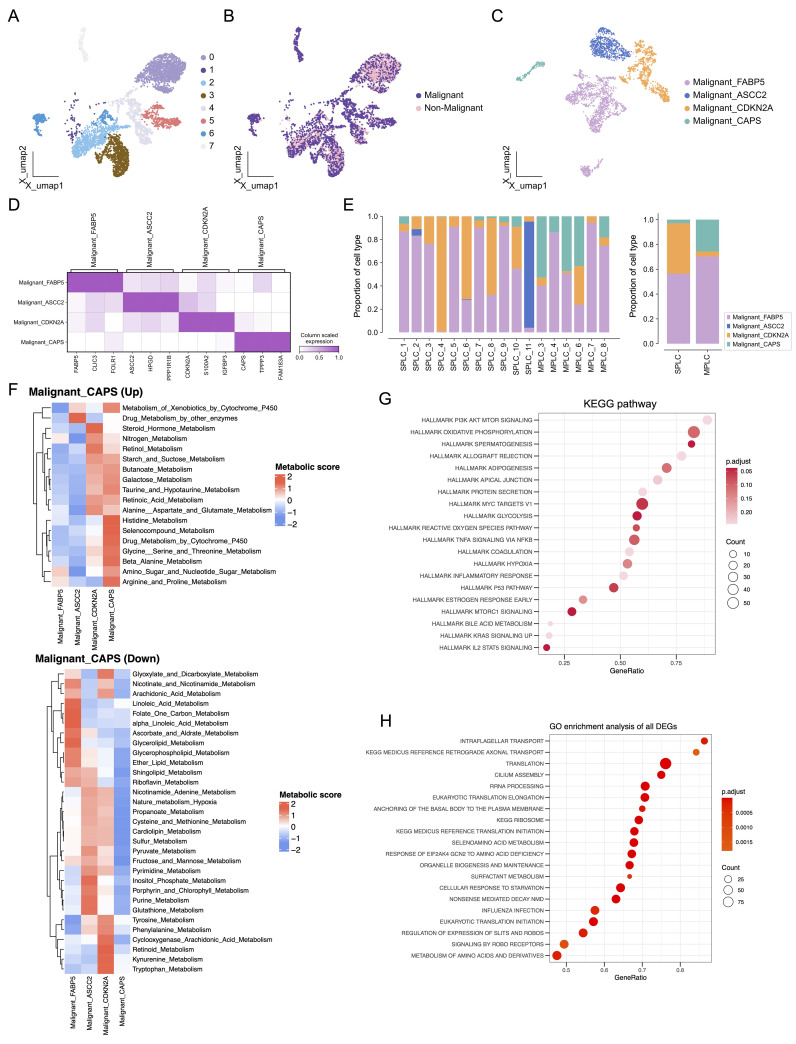
Malignant epithelial cell heterogeneity and metabolic reprogramming in MPLC and SPLC. **(A)** UMAP projection of epithelial cells partitioned into 8 transcriptionally distinct clusters. **(B)** Separation of malignant (high CNV score) and non-malignant (low CNV score) epithelial cells on UMAP. **(C)** Re-clustering of malignant epithelial cells into 4 subpopulations. **(D)** Heatmap showing expression levels of marker genes defining each malignant subpopulation. **(E)** Stacked bar plots depicting the proportion of malignant subpopulations across individual samples and grouped by SPLC/MPLC. **(F)** Heatmap of normalized metabolic pathway activity scores in the Malignant_CAPS versus other malignant subpopulations. Color intensity reflects pathway enrichment scores. **(G, H)** Enrichment plots of KEGG pathways **(G)** and GO enrichment analysis **(H)** in Malignant_CAPS.

Epithelial cells were analyzed to delineate tumor-specific differences between MPLC and SPLC. Since the MPLC_1 and MPLC_2 samples were derived from a squamous cell carcinoma patient, they were excluded from this analysis to avoid confounding effects from histological subtype. Therefore, all subsequent epithelial and malignant subpopulation analyses were conducted in an adenocarcinoma-focused context. Epithelial cells were clustered into eight distinct groups (**Figure 2A**). Several clusters exhibited patient-specific enrichment, such as cluster 0 predominantly in SPLC_11 and cluster 3 in SPLC_9, whereas clusters 1, 2, and 4 were more evenly distributed across samples (**Supplementary Figure 2A**). Using T and NK cells as a reference, we inferred chromosomal copy-number variations (CNVs) in epithelial cells and discriminated 3, 275 malignant cells from 1, 385 non-malignant cells (**Figure 2B**). Notably, SPLC samples showed a higher proportion of non-malignant epithelial cells compared to MPLC (**Supplementary Figures 2B, C**). All malignant epithelial cells were further grouped into four subpopulations, named according to their top marker genes. Among these, the Malignant_FABP5 subgroup was the most abundant (1, 473 cells, 44.98%), followed by Malignant_CDKN2A (868 cells, 26.50%) (**Figure 2C**). Each malignant subgroup displayed unique marker gene expression patterns (**Figure 2D**).

We then visualized the distribution of these malignant subpopulations across individual samples. The Malignant_ASCC2 subgroup was mainly restricted to SPLC_11, reflecting marked inter-patient heterogeneity (**Figure 2E**). To minimize bias, SPLC_11 was excluded from group-wise comparisons. After exclusion, we found that Malignant_FABP5 remained the predominant subgroup in both MPLC and SPLC. Malignant_CDKN2A was enriched in SPLC, while Malignant_CAPS, although relatively rare overall, was consistently more prevalent in MPLC (**Figure 2E**). This enrichment was further supported by a higher proportion of Malignant_CAPS cells in MPLC compared to SPLC samples (p = 0.0168, Wilcoxon rank-sum test, **Supplementary Figure 2D**), suggesting it represents an MPLC-enriched malignant epithelial cell subpopulation. The Malignant_CAPS subgroup was characterized by selective enrichment of calcyphosin (CAPS), tubulin polymerization promoting protein family member 3 (TPPP3), and family with sequence similarity 182 member A (FAM182A), genes associated with epithelial differentiation and cilium-related cellular organization.

Recent studies have highlighted the close link between metabolic heterogeneity within the tumor microenvironment and immune regulation. To investigate this in the context of MPLC, we performed a comparative enrichment analysis of metabolism-related pathways in the MPLC-enriched Malignant_CAPS versus other malignant subpopulations. We found that Malignant_CAPS exhibited significant upregulation of several metabolic pathways, including histidine metabolism, glycine, serine and threonine metabolism, beta-alanine metabolism, arginine and proline metabolism, selenocompound metabolism, and amino sugar and nucleotide sugar metabolism (**Figure 2F**). These pathways are involved in amino acid and redox metabolism and have been implicated in supporting rapid cell proliferation and modulating immune cell function in the tumor microenvironment. Consistently, single-cell metabolic activity analysis further supported increased activity of arginine and proline metabolism and histidine metabolism in the Malignant_CAPS subpopulation (**Supplementary Figure 2E**). Conversely, pathways such as nicotinamide adenine metabolism, hypoxia-related metabolism, propanoate metabolism, sulfur metabolism, cysteine and methionine metabolism, and cardiolipin metabolism were downregulated in this subpopulation, suggesting a metabolic shift towards anabolic processes and possibly reduced dependence on hypoxic adaptation (**Figure 2F**). In addition, hallmark pathway analysis revealed enrichment of PI3K/AKT/mTOR signaling, glycolysis, oxidative phosphorylation, hypoxia, and inflammatory response (**Figure 2G**). Many of these pathways are known to drive metabolic reprogramming, promote tumor cell survival, and interact with immune regulatory networks. Furthermore, KEGG pathway enrichment analysis highlighted the biosynthetic and structural features of Malignant_CAPS. Pathways related to protein translation, ribosome function and cilium assembly were significantly enriched, indicating high synthetic activity and cellular reorganization in this subpopulation (**Figure 2H**). Gene Ontology (GO) enrichment analysis revealed similar results, with Malignant_CAPS significantly enriched in biological processes related to protein localization and targeting, cilium assembly as well as RNA degradation and translation initiation (**Supplementary Figure 2F**).

### 3.3 Enrichment of immunosuppressive CD4^+^ T cell subpopulations in MPLC

**Figure 3:**
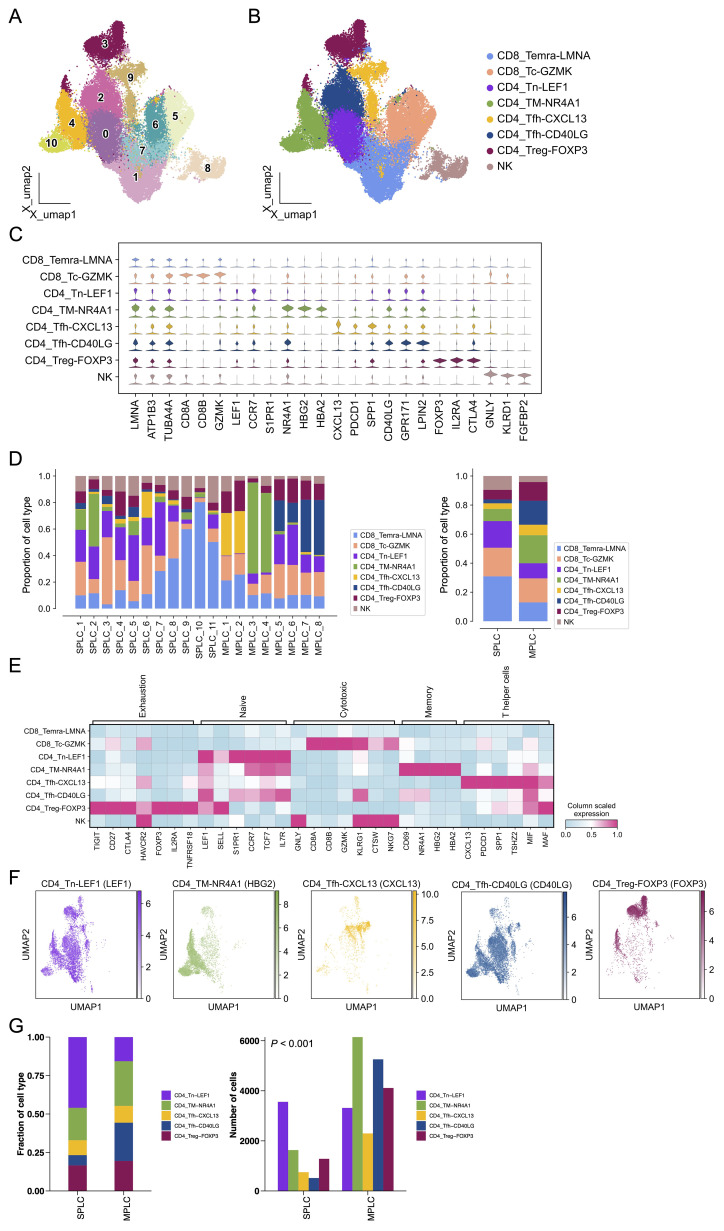
T/NK cell heterogeneity and CD4^+^ T cell subpopulation disparities between MPLC and SPLC. **(A)** UMAP visualization of T/NK cells clustered into 11 transcriptionally distinct subsets. **(B)** Annotation of T/NK subsets: 2 CD8^+^ T cell, 5 CD4^+^ T cell, and 1 NK cell population, labeled by canonical markers. **(C)** Violin plots illustrating the expression distribution of key marker genes in each T/NK subpopulation. **(D)** Stacked bar plot showing the proportion of each T/NK subpopulation across individual samples and comparative analysis of T/NK subpopulation proportions between SPLC and MPLC groups. **(E)** Refined annotation of CD4^+^ T cell subpopulations based on functional marker expression. **(F)** UMAP visualization of marker gene expression levels for key CD4^+^ T cell subpopulations. **(G)** Group-wise comparison of CD4^+^ T cell subset proportions (MPLC vs. SPLC), emphasizing enriched populations in MPLC.

To characterize T and NK cell heterogeneity in MPLC and SPLC, we performed re-clustering of T/NK cells, the most abundant immune population in our dataset. This analysis identified 11 distinct clusters (**Figure 3A**), which were annotated into eight major subpopulations based on canonical marker gene expression (**Figures 3B, C**). These included two CD8^+^ T cell subtypes, five CD4^+^ T cell subtypes, and a single NK cell population. We next compared the distribution of these subpopulations across samples. CD8^+^ T cells showed relatively consistent proportions between samples, whereas CD4^+^ T cells exhibited substantial inter-sample variability. Specifically, SPLC samples displayed a higher proportion of CD8_Temra-LMNA and CD4_Tn-LEF1. In contrast, MPLC samples were enriched in multiple CD4^+^ T cell subtypes, including immunosuppressive and helper phenotypes (**Figure 3D**). We further annotated CD4^+^ T cell subpopulations based on the expression of key functional markers (**Figure 3E**). Among these, CD4_Treg-FOXP3 showed high expression of canonical regulatory T cell markers, indicating a strong immunosuppressive or exhausted phenotype. In contrast, CD4_Tn-LEF1, which was more prevalent in SPLC, corresponded to naïve CD4^+^ T cells. Marker gene expression patterns of CD4^+^ T cell subpopulations are shown in **Figure 3F**. Notably, MPLC samples contained markedly higher proportions of CD4_Treg-FOXP3 and lower proportions of CD4_Tn-LEF1 compared to SPLC (**Figure 3G**). These findings highlight the prominent enrichment of immunosuppressive CD4^+^ T cell subpopulations in MPLC, supporting the notion of a more immunosuppressive tumor microenvironment in MPLC compared to SPLC.

### 3.4 Myeloid cell heterogeneity and interaction patterns between MPLC and SPLC

**Figure 4:**
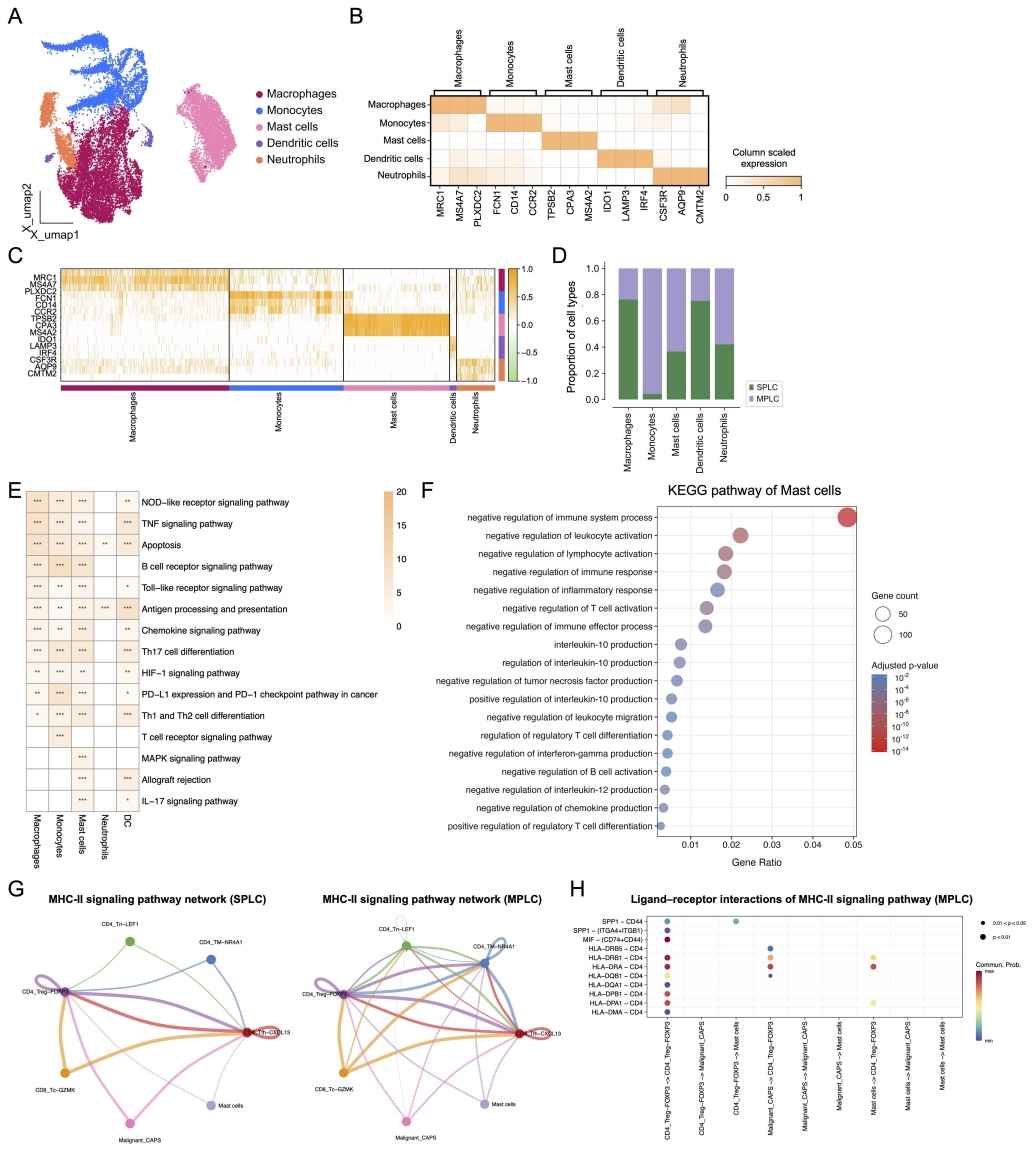
Myeloid cell landscape and intercellular crosstalk in MPLC versus SPLC. **(A)** UMAP plot of myeloid cells color-coded by 5 annotated subpopulations. **(B)** Column-scaled expression heatmap of marker genes (rows) across five myeloid subpopulations (columns), demonstrating distinct transcriptional profiles. Color intensity represents z-score normalized expression levels. **(C)** Heatmap displaying single-cell expression levels of key marker genes (x-axis) across myeloid subtypes (y-axis). Color gradient reflects log-normalized expression values. **(D)** Stacked bar plot showing the proportional distribution of five myeloid subpopulations between SPLC and MPLC groups. **(E)** Heatmap of pathway enrichment scores for marker genes from different myeloid subpopulations. **(F)** Dot plot showing enriched pathways for mast cell-specific marker genes. Dot size represents gene ratio and color indicates -log10(adjusted p-value). **(G)** Cell-cell interaction network for MHC-II signaling pathway, showing interactions between Malignant_CAPS, CD4^+^ T cells and mast cells. Line thickness represents interaction strength. **(H)** Dot plot of significant ligand-receptor pairs involved in MHC-II signaling in MPLC. Dot size represents p-value range and color intensity indicates communication probability. *adjusted p-value < 0.05, **adjusted p-value < 0.01, and ***adjusted p-value < 0.001.

We next examined the myeloid compartment to compare its composition and potential immune-regulatory roles in MPLC and SPLC. All myeloid cells were clustered into 21 distinct groups (**Supplementary Figure 3A**), the majority of which showed comparable distributions between MPLC and SPLC, while a subset of clusters exhibited preferential enrichment in MPLC (**Supplementary Figure 3B**). Based on marker gene expression, these clusters were consolidated into five major myeloid cell types: macrophages, dendritic cells, mast cells, monocytes, and neutrophils (**Figure 4A**), each exhibiting distinct marker profiles (**Figures 4B, C**; **Supplementary Figure 3C**). We observed that macrophages and dendritic cells were relatively enriched in SPLC samples, whereas mast cells and monocytes were more abundant in MPLC (**Figure 4D**). This shift may reflect a more immunoregulatory and suppressive microenvironment in MPLC, contrasting with the inflammatory features associated with SPLC.

Pathway enrichment analysis of marker genes from different myeloid subpopulations revealed distinct immune-related functional profiles. Notably, mast cells demonstrated the most extensive enrichment across both immune activation and immune regulatory pathways, suggesting a potential dual role of mast cells in orchestrating immune responses within the tumor microenvironment (**Figure 4E**). To further characterize the immunoregulatory potential of mast cells, we performed a focused enrichment analysis of mast cell-pecific marker genes. This analysis revealed a predominant enrichment in immunosuppressive-related biological processes, including negative regulation of immune system process, negative regulation of lymphocyte and T cell activation, negative regulation of inflammatory and immune effector responses. Notably, mast cells were also associated with interleukin-10 production, positive regulation of regulatory T cell differentiation, and inhibition of pro-inflammatory cytokines (TNF, IFN-γ, IL-12), highlighting their potential as key mediators of immune suppression (**Figure 4F**). Taken together, these findings suggest that mast cells represent a unique myeloid subpopulation with a multifaceted role in integrating both immune-activating and immune-inhibitory signals, with a dominant tendency toward immunosuppressive regulation.

To further elucidate the immunomodulatory network in MPLC, we analyzed the cell-cell interactions between the MPLC-enriched Malignant_CAPS, CD4^+^ T cell subpopulations, and mast cells. We focused on the MHC-II signaling pathway because it exhibited distinct interaction patterns between MPLC and SPLC, consistent with its known role in immune regulation (**Figure 4G**; **Supplementary Figure 3D**). In SPLC, Malignant_CAPS primarily interacted with the immunosuppressive CD4_Treg-FOXP3 and helper CD4_Tfh-CXCL13 subpopulations, forming a focused and strong interaction network. In contrast, in MPLC, Malignant_CAPS engaged with a broader range of CD4^+^ T cell subsets, including CD4_Tn-LEF1 and CD4_TM_NR4A1, although the overall interaction strength was weaker. This pattern suggests that MPLC tumors may induce a more complex yet less efficient immunoregulatory network. Additionally, the interactions among CD4^+^ T cell subpopulations themselves were stronger and more extensive in MPLC, indicating a reorganization of the T cell network toward a more interconnected state. Finally, mast cells in MPLC exhibited markedly enhanced interactions with CD4^+^ T cells compared to SPLC, supporting their unique role in shaping an immunosuppressive microenvironment. These findings were further supported by ligand-receptor interaction analysis of the MHC-II signaling pathway in MPLC, which highlighted specific interactions among Malignant_CAPS, CD4_Treg-FOXP3, and mast cells mediated through HLA class II-related signaling involving HLA-DRA and CD44 (**Figure 4H**).

### 3.5 Metabolism-based prognostic model in LUAD derived from the malignant_CAPS subpopulation

**Figure 5:**
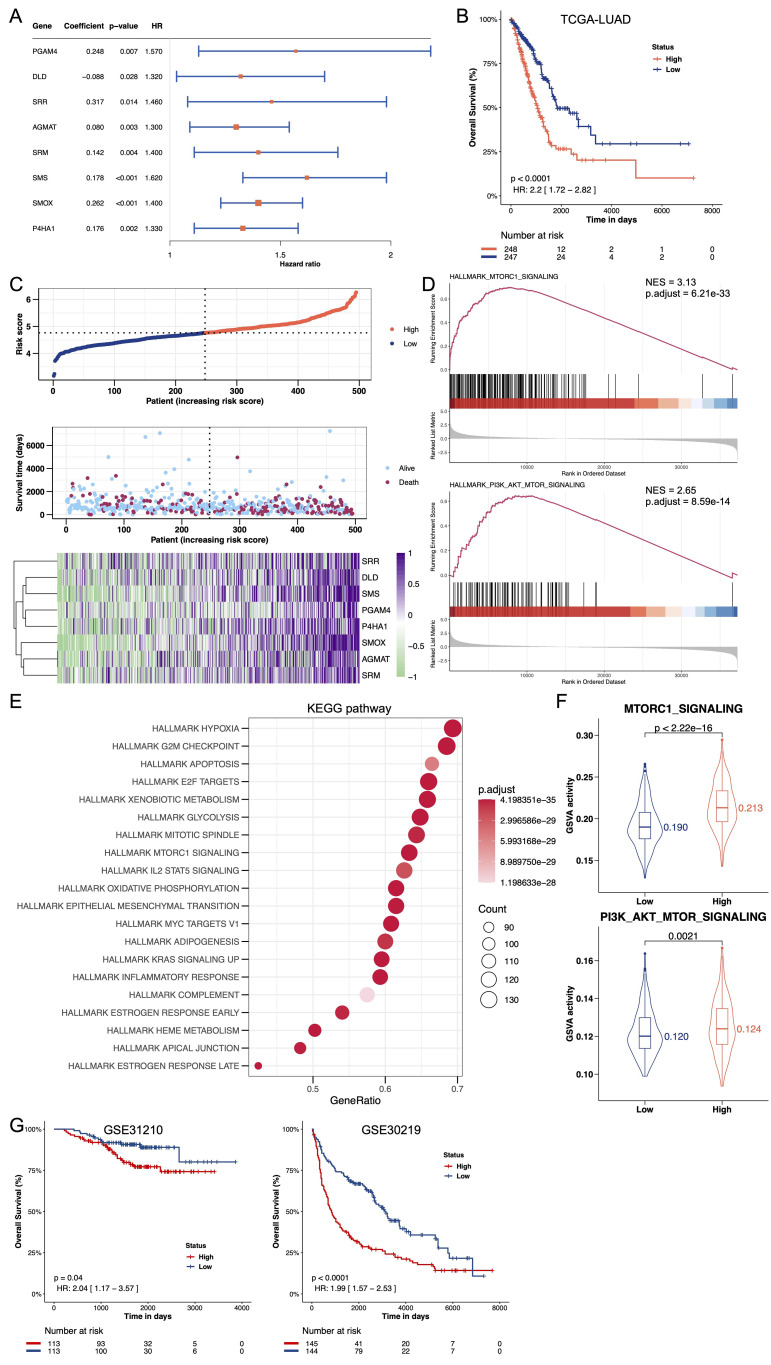
Metabolic risk signature from malignant_CAPS subpopulation predicts LUAD prognosis. **(A)** Forest plot displaying the Cox regression results of prognostic signature genes, showing coefficients, hazard ratios (HRs) with 95% confidence intervals, and corresponding p-values. **(B)** Kaplan-Meier survival analysis in TCGA-LUAD cohort (training set) stratified by median risk score. **(C)** Risk assessment plot showing (from top to bottom): risk score distribution (blue: low-risk, orange: high-risk), survival status (dots) and time (y-axis) for each patient, heatmap of signature gene expression patterns across patients. **(D)** GSEA enrichment plot highlighting significant activation of HALLMARK_MTORC1_SIGNALING pathway in high-risk group. **(E)** Bubble plot of hallmark pathway enrichment analysis for genes upregulated in high-risk group. Bubble size represents gene ratio and color indicates -log10(adjusted p-value). **(F)** Violin plots comparing single-sample GSVA scores for MTORC1_SIGNALING and PI3K_AKT_MTOR_SIGNALING pathways between risk groups. **(G)** External validation of the prognostic model in independent GEO datasets (GSE31210 left, GSE30219 right), showing consistent survival stratification.

Given the unique metabolic features of the MPLC-enriched Malignant_CAPS subpopulation and its interactions with immunosuppressive CD4^+^ T cells and mast cells, we hypothesized that this subpopulation might regulate patient prognosis through metabolic reprogramming and immune modulation. To test this hypothesis, we selected gene sets from the significantly upregulated metabolic pathways in Malignant_CAPS, including arginine and proline metabolism, beta-alanine metabolism, histidine metabolism, selenocompound metabolism, and glycine, serine and threonine metabolism, and constructed a LUAD prognostic model using bulk RNA-seq data. Candidate genes were first screened by univariate Cox regression and subsequently subjected to LASSO Cox regression with 10-fold cross-validation to prevent overfitting. The optimal penalty parameter was determined based on the minimum cross-validation error (lambda.min) (**Supplementary Figure 4A**), and the coefficient shrinkage process is shown in **Supplementary Figure 4B**. Using this approach, we identified an eight-gene prognostic signature (**Figure 5A**): Risk score = (0.248 × phosphoglycerate mutase family member 4 (PGAM4)) + (–0.088 × phosphoglycerate kinase 1 (PGK1)) + (0.317 × serine racemase (SRR)) + (0.080 × agmatinase (AGMAT)) + (0.142 × SR spermidine synthase (SRM)) + (0.178 × SMS) + (0.262 × SMOX) + (0.176 × prolyl 4-hydroxylase subunit alpha 1 (P4HA1)). In the TCGA-LUAD training cohort, the model effectively stratified patients into high- and low-risk groups based on the median risk score. High-risk patients showed significantly worse overall survival (p < 0.0001, HR = 2.20 [1.72-2.82]; **Figure 5B**). The model remained an independent predictor after adjusting for clinical factors (**Supplementary Figure 4C**). Visualization of individual patient data showed a gradual increase in model gene expression and risk score from left to right, accompanied by higher mortality rates (**Figure 5C**). Further expression analysis showed that six genes in the model, AGMAT, P4HA1, PGAM4, SMOX, SMS, and SRM were significantly upregulated in tumor tissues compared to adjacent normal tissues, whereas SRR exhibited higher expression in normal samples (**Supplementary Figure 4D**). To explore the biological relationships among the model genes, we constructed a protein-protein interaction (PPI) network using STRING (**Supplementary Figure 4E**). Notably, AGMAT, SRM, SMS, and SMOX were highly interconnected, suggesting functional coordination within the arginine and proline metabolism and glycine, serine and threonine metabolism pathways (**Supplementary Figure 4F**). Mechanistically, gene set enrichment analysis (GSEA) revealed that the high-risk group was enriched in pathways such as PI3K/AKT/mTOR signaling, which is consistent with the molecular characteristics of the Malignant_CAPS subpopulation. Furthermore, pathway enrichment analysis of genes upregulated in the high-risk group revealed significant enrichment of metabolic reprogramming pathways, including glycolysis, oxidative phosphorylation, and adipogenesis, as well as the inflammatory response pathway, supporting that the metabolic and signaling characteristics of the high-risk group originate from the Malignant_CAPS subpopulation. Additional enrichment in cell cycle-elated pathways (G2M checkpoint, MYC targets, E2F targets) and the epithelial esenchymal transition (EMT) pathway further suggests increased proliferative and invasive potential in high-risk tumors (**Figures 5D, E**). This was supported by single-sample GSVA analysis, which also confirmed significantly higher activity of PI3K/AKT/mTOR and mTORC1 signaling in the high-risk group (**Figure 5F**). Finally, we validated the prognostic performance of the model in two independent lung adenocarcinoma cohorts (GSE31210 and GSE30219). In both external datasets, high-risk patients exhibited significantly worse survival than low-risk patients, demonstrating the robustness and generalizability of the model (**Figure 5G**).

### 3.6 Immune landscape and immunotherapy implications of the risk groups

**Figure 6:**
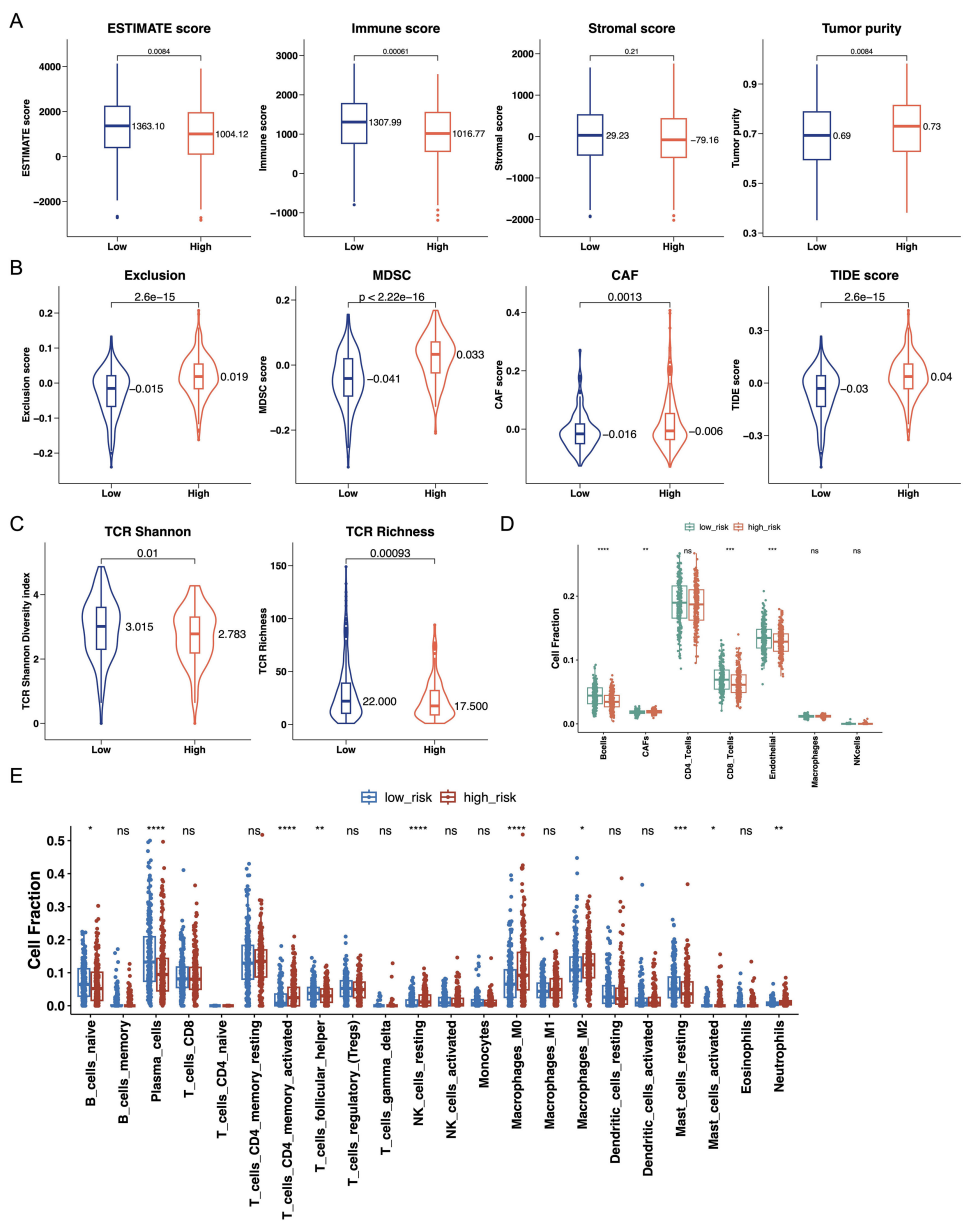
Distinct immune microenvironment characteristics between metabolic risk groups. **(A)** Box plots comparing ESTIMATE-derived immune and stromal scores between high- and low-risk groups. **(B)** Violin plots demonstrate immune exclusion signature profiling between risk groups. **(C)** Violin plots showing significant differences in TCR Shannon diversity and TCR richness between high- and low-risk groups, indicating variation in T cell receptor diversity. **(D)** Box plots displaying the estimated proportions of seven immune/stromal cell types calculated by the EPIC algorithm, highlighting compositional differences in the tumor microenvironment between the two risk groups. **(E)** Comparative bar plot showing CIBERSORT-inferred immune cell proportions between high- and low-risk groups. ns, not significant; *adjusted p-value < 0.05, **adjusted p-value < 0.01, ***adjusted p-value < 0.001, and ****adjusted p-value < 0.0001.

We next compared immune-related features between the high- and low-risk groups to explore differences in the tumor microenvironment. The low-risk group exhibited significantly higher ESTIMATE and Immune scores, indicating greater immune cell infiltration, and correspondingly lower tumor purity compared to the high-risk group (**Figure 6A**). In contrast, the high-risk group showed significantly higher Exclusion, MDSC, and CAF scores, reflecting enhanced immune suppression, stromal remodeling, and immune evasion potential; notably, the higher TIDE scores observed in this group further imply a reduced likelihood of responding to immune checkpoint inhibitor therapy, whereas the low-risk group may derive greater benefit (**Figure 6B**).

We also observed that the low-risk group had significantly higher TCR Shannon diversity and TCR richness (**Figure 6C**), suggesting a more diverse and abundant T cell receptor repertoire, which is indicative of a more active and responsive adaptive immune response. To further dissect immune microenvironment differences, we deconvoluted bulk RNA-seq data using CIBERSORT and EPIC algorithms. EPIC-based deconvolution further confirmed that B cells and CD8^+^ T cells were significantly more abundant in the low-risk group (**Figure 6D**), indicating stronger cytotoxic immune responses. CIBERSORT analysis revealed that the high-risk group was significantly enriched in activated memory CD4^+^ T cells, M0 and M2 macrophages, activated mast cells, and neutrophils. These cell types are commonly associated with chronic inflammation and immunosuppressive tumor environments (**Figure 6E**). These findings indicate that the high-risk group is characterized by reduced immune infiltration, impaired T cell diversity, and increased immunosuppressive and stromal features, consistent with the metabolic immunomodulatory properties of the Malignant_CAPS subpopulation identified in the single-cell analysis.

### 3.7 Drug sensitivity analysis in high- and low-risk groups

**Figure 7:**
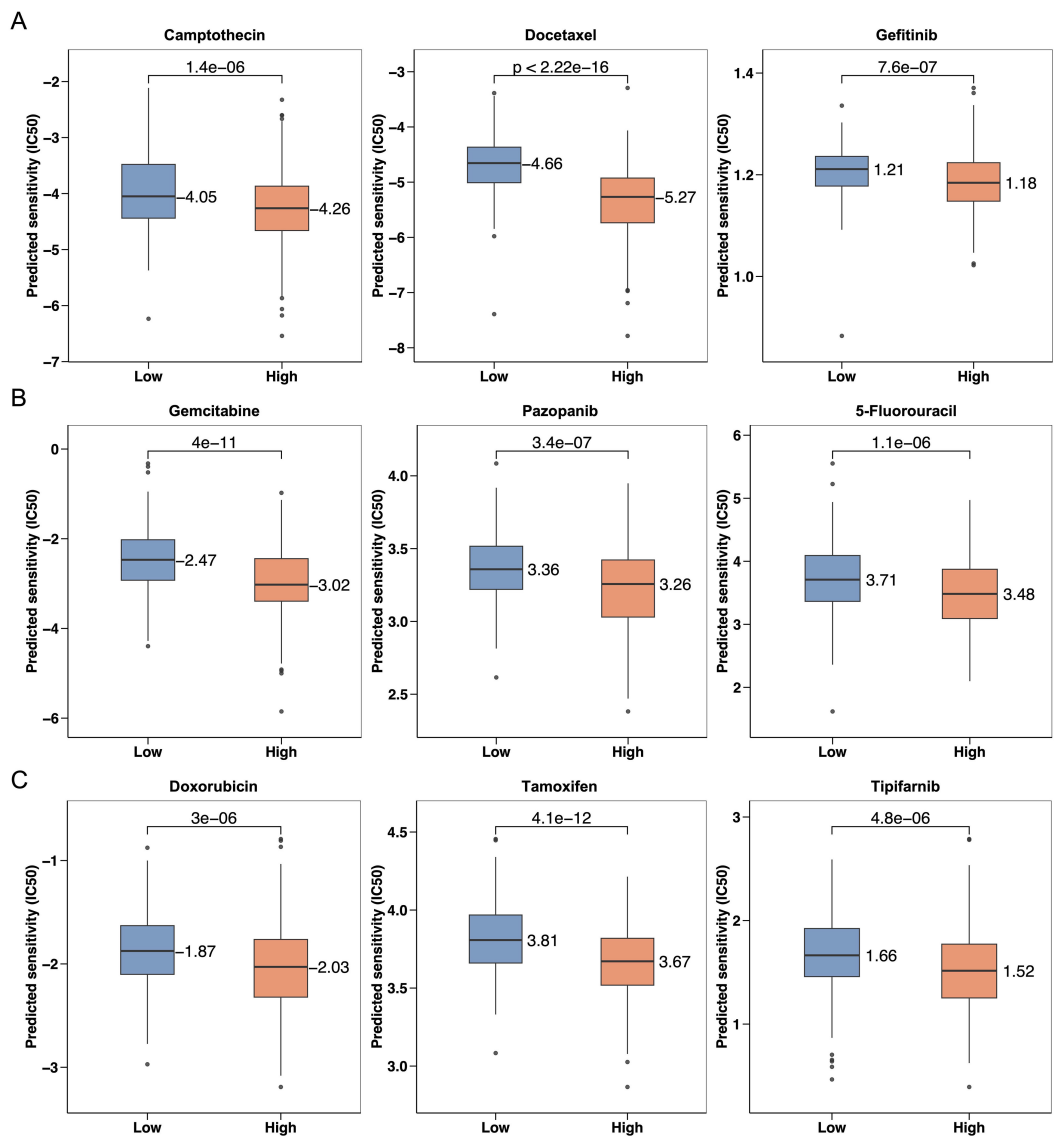
Drug sensitivity patterns in metabolic risk groups. Box plots show group comparisons of predicted sensitivity (IC50) to multiple therapeutic agents: topoisomerase inhibitors (camptothecin), microtubule inhibitors (docetaxel), EGFR inhibitors (gefitinib) **(A)**; antimetabolites (gemcitabine, 5-fluorouracil) and multi-kinase inhibitors (pazopanib) **(B)**; and DNA intercalators (doxorubicin), SERMs (tamoxifen), and farnesyltransferase inhibitors (tipifarnib) **(C)**.

We further assessed drug sensitivity in cell lines stratified by the risk model to explore potential therapeutic vulnerabilities associated with the risk model. The high-risk group showed significantly greater sensitivity to a panel of chemotherapeutic and targeted agents, including the topoisomerase inhibitor camptothecin, the microtubule inhibitor docetaxel, the EGFR tyrosine kinase inhibitor gefitinib (**Figure 7A**), the nucleoside analog gemcitabine, the multi-kinase inhibitor pazopanib, the antimetabolite 5-fluorouracil (**Figure 7B**), the anthracycline doxorubicin, the selective estrogen receptor modulator tamoxifen, and the farnesyltransferase inhibitor tipifarnib (**Figure 7C**). These agents target key processes such as DNA replication, cell cycle progression, metabolic pathways, and oncogenic signaling, which are highly active in the metabolically reprogrammed and proliferative tumors of the high-risk group. Given the immunosuppressive and exclusionary tumor microenvironment observed in high-risk patients, these findings suggest that high-risk tumors may exhibit increased susceptibility to selected cytotoxic or targeted therapeutic strategies, warranting further experimental investigation.

### 3.8 Functional validation of SMOX and SMS in tumor cell proliferation, invasion, and PI3K/mTOR-related transcriptional changes

**Figure 8:**
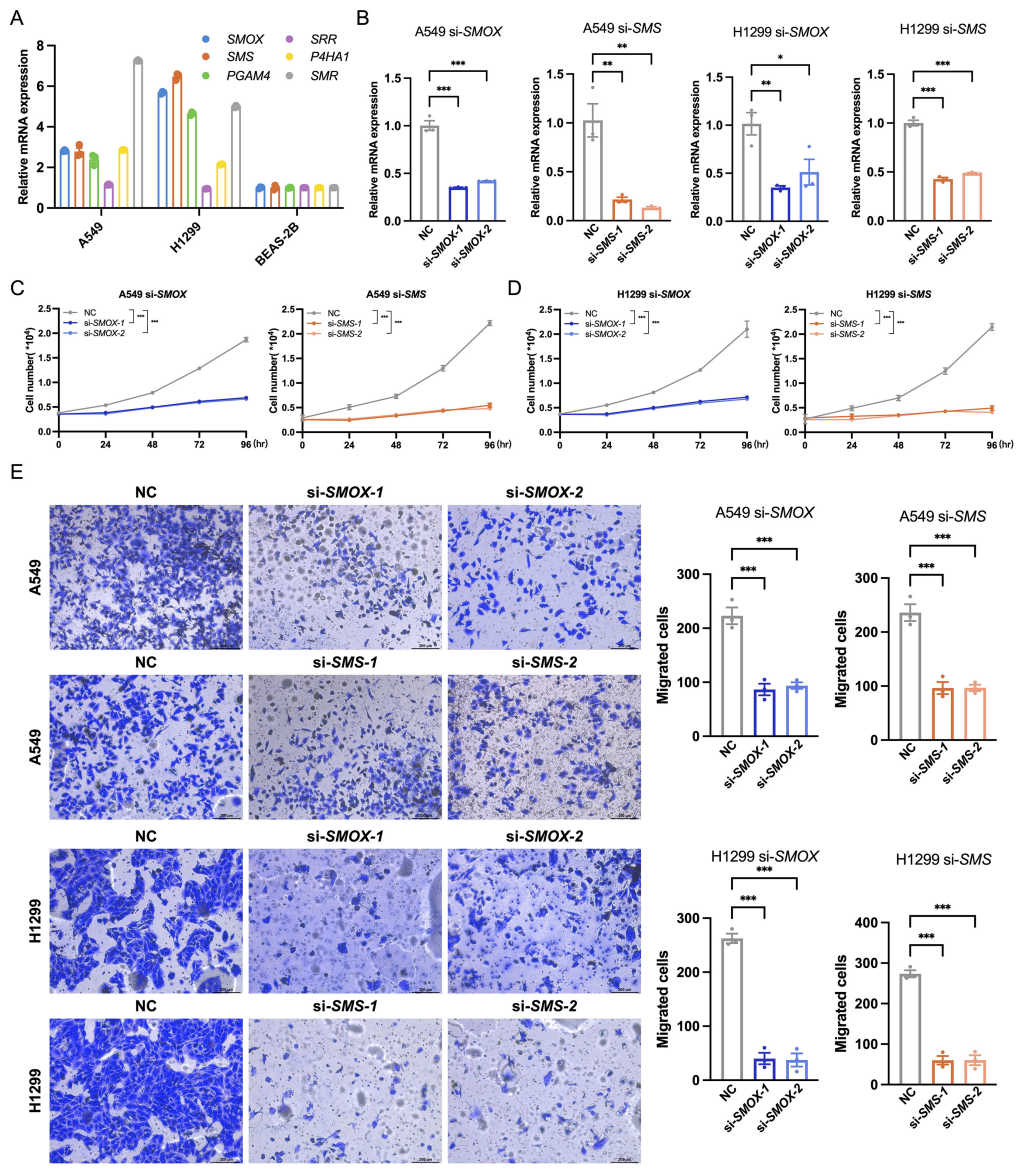
Expression and functional validation of model genes SMOX and SMS in lung cancer cell lines. **(A)** mRNA expression levels of model genes (SMOX, SMS, SRM, PGAM1, P4HA1, SRR) in normal cell line BEAS-2B and lung cancer cell lines A549 and H1299. **(B)** qPCR validation of siRNA-mediated knockdown efficiency for SMOX and SMS in A549 and H1299 cells. **(C, D)** Cell proliferation was significantly inhibited upon SMOX or SMS knockdown, as assessed by CCK-8 assay in A549 **(C)** and H1299 **(D)** cells. **(E)** Transwell migration assay showing reduced migratory capacity following SMOX or SMS knockdown in both cell lines. Data are presented as mean ± SD; *P < 0.05, **P < 0.01, ***P < 0.001.

**Figure 9:**
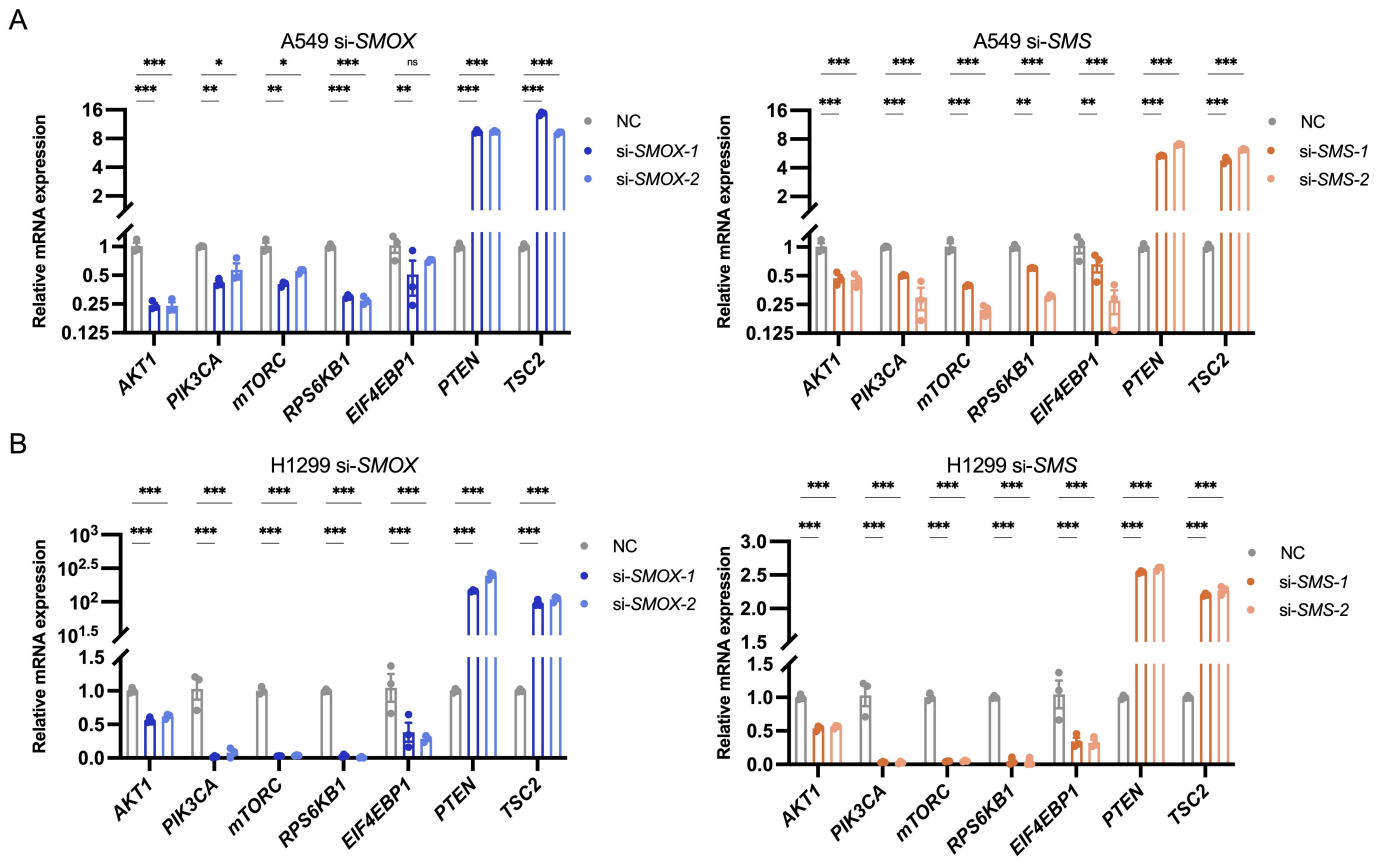
SMOX and SMS modulate PI3K/mTOR signaling pathway in lung cancer cells. **(A, B)** Quantitative PCR analysis of key components in the PI3K/mTOR signaling pathway following SMOX or SMS knockdown in A549 **(A)** and H1299 **(B)** cells. Data are presented as mean ± SD, with *P < 0.05, **P < 0.01, and ***P < 0.001.

To experimentally validate the expression and functional roles of model genes, we first assessed their mRNA levels in two lung cancer cell lines (A549 and H1299) and the normal bronchial epithelial cell line BEAS-2B. Except for SRR, all model genes were significantly upregulated in cancer cell lines, with SMOX, SMS, and SRM showing the most pronounced overexpression (**Figure 8A**). We prioritized SMOX and SMS for functional validation based on their central roles in the polyamine metabolic pathway. We performed siRNA-mediated knockdown of SMOX and SMS in A549 and H1299 cells, and confirmed efficient silencing of both genes compared to negative controls (**Figure 8B**). CCK-8 assays revealed that knockdown of SMOX and SMS markedly reduced cell proliferation rates in both cell lines (**Figures 8C, D**). In parallel, transwell migration assays demonstrated significantly impaired migratory capacity following SMOX or SMS knockdown, as evidenced by reduced numbers of migrated cells compared to the control group (**Figure 8E**). These findings indicate that SMOX and SMS are critical for maintaining the proliferative potential and promoting the migratory ability of lung cancer cells. To explore potential downstream signaling effects, we next assessed the expression of key genes in the PI3K-mTOR pathway following SMOX and SMS silencing. qPCR analysis showed that individual knockdown of SMOX or SMS resulted in downregulation of phosphatidylinositol-4, 5-bisphosphate 3-kinase catalytic subunit alpha (PIK3CA), AKT serine/threonine kinase 1 (AKT1), and core mTORC1 effectors including rapamycin kinase (MTOR), ribosomal protein S6 kinase B1 (RPS6KB1), and eukaryotic translation initiation factor 4E binding protein 1 (EIF4EBP1), while the negative regulators phosphatase and tensin homolog (PTEN) and tuberous sclerosis complex 2 (TSC2) were significantly upregulated (**Figures 9A, B**). These results suggest that SMOX and SMS may be associated with transcriptional regulation of the PI3K/mTOR signaling pathway, providing a mechanistic link between polyamine metabolism and oncogenic signaling in high-risk lung cancer.

## 4 Discussion

In this study, we performed an integrative single-cell and bulk transcriptomic analysis to uncover the molecular and immunological features of MPLC. We identified a MPLC-enriched malignant subpopulation, termed Malignant_CAPS, characterized by distinct metabolic reprogramming and immunoregulatory interactions with the tumor microenvironment. This subpopulation exhibited upregulation of several amino acid-related metabolic pathways and showed enhanced ligand-receptor communication with immunosuppressive CD4^+^ T cells and mast cells. Based on these subtype-specific metabolic features, we constructed a robust LUAD prognostic model that stratifies patient risk and correlates with immune infiltration, tumor immune evasion, and therapeutic response. These findings suggest that metabolic-immune crosstalk may be a key driver of poor prognosis and offer a basis for developing metabolism-informed therapeutic strategies in lung adenocarcinoma.

Recent studies have highlighted that tumor-intrinsic transcriptional and metabolic reprogramming plays a crucial role in shaping the immune microenvironment, particularly by modulating immune cell recruitment, activation, and function. In our study, the MPLC-enriched Malignant_CAPS subpopulation also showed selective enrichment of TPPP3 and FAM182A, genes associated with epithelial differentiation and cilium-related cellular organization. Notably, TPPP3 has been implicated in microtubule stabilization, ciliogenesis, and malignant progression in NSCLC (31–33). Consistent with our findings, recent single-cell studies of lung adenocarcinoma have identified epithelial trajectories characterized by cilium movement, microtubule bundle formation, and increased TPPP3 expression during malignant progression (34), supporting the biological relevance of the epithelial remodeling features observed in Malignant_CAPS. In parallel, the MPLC-enriched Malignant_CAPS cells exhibited upregulation of multiple metabolic pathways, including histidine, glycine/serine/threonine, arginine/proline, and selenocompound metabolism, which may collectively contribute to immune suppression and evasion. For instance, increased histidine catabolism has been shown to promote immunosuppressive myeloid cell differentiation and impair antitumor T cell responses in lung adenocarcinoma (35). Elevated glycine and serine metabolism fuels nucleotide biosynthesis and supports the survival of regulatory T cells (Tregs), further favoring immune evasion (36, 37). Similarly, arginine depletion through upregulated ARG1 or SAT1 in tumor cells can suppress T cell proliferation and cytotoxicity, a mechanism well documented in NSCLC and other solid tumors (38). Interestingly, hallmark analysis also revealed concurrent enrichment of oxidative phosphorylation and glycolysis in the Malignant_CAPS subpopulation. Although oxidative phosphorylation is a fundamental metabolic process in most cells, its simultaneous activation together with glycolysis has increasingly been recognized as a feature of metabolically flexible tumor cells rather than simply reflecting basal mitochondrial activity. Consistent with previous studies demonstrating substantial metabolic heterogeneity in lung cancer and metabolic divergence among different MPLC lesions, suggesting that the Malignant_CAPS subpopulation represents a metabolically reprogrammed epithelial state with enhanced adaptability to heterogeneous tumor microenvironments (39, 40). Conversely, several immunologically supportive metabolic pathways were downregulated in the malignant CAPS cluster, including NAD^+^ biosynthesis and sulfur/methionine metabolism. Notably, NAD^+^ is essential for T cell mitochondrial function and activation, and its depletion in the tumor milieu has been associated with impaired CD8^+^ T cell responses (41, 42). Hypoxia-related metabolic suppression may also reduce the efficacy of immune recognition, as hypoxia-inducible factors are critical in sustaining antigen presentation and effector T cell function under stress (43).

In addition to metabolic alterations, our study revealed enhanced MHC-II-mediated interactions between malignant cells and immune populations, particularly CD4^+^ T cells and mast cells. Tumor cell-intrinsic MHC-II expression has been implicated in modulating helper T cell responses and facilitating immunoediting. For instance, recent findings in lung cancer have shown that MHC-II+ tumor epithelial cells can directly present antigens to CD4^+^ T cells, driving either antitumor immunity or tolerance depending on the cytokine milieu (44, 45). Moreover, mast cells interacting with tumor cells via antigen presentation or chemokine gradients may contribute to chronic inflammation and immunosuppressive remodeling (46, 47). These metabolic and immunological features of the CAPS subpopulation may jointly define a tumor-promoting phenotype, driving poor prognosis and resistance to immune checkpoint therapies. However, we note that the current findings are primarily based on transcriptomic inference, and definitive mechanistic validation will be required to further elucidate these interactions.

In our study, several prognostic genes, MOX, SMS, SRM, PGAM1, and P4HA1 were predominantly involved in polyamine metabolism, glycolysis, and collagen remodeling, and were consistently upregulated in high-risk tumors. Recent studies have shown that dysregulation of these genes is closely associated with immune modulation, tumor proliferation, and poor prognosis in lung cancer. SMOX is frequently overexpressed in NSCLC tumors and associated with poorer survival, potentially due to its production of ROS that can drive DNA damage and an inflammatory, immunosuppressive microenvironment (48, 49). SMS overexpression in LUAD correlates with advanced stage and worse prognosis, and functional knockdown of SMS significantly impairs tumor cell proliferation and invasion (50). Likewise, the glycolytic enzyme PGAM1 is a downstream effector of oncogenic PI3K/mTOR signaling: mTOR HIF1α-driven PGAM1 upregulation sustains the Warburg effect, and high PGAM1 levels promote NSCLC cell proliferation and tumorigenesis, correlating with shortened survival (51). Meanwhile, P4HA1, a hypoxia-inducible collagen prolyl-4-hydroxylase, emerges as a potent driver of metastasis and chemoresistance in lung adenocarcinoma; its overexpression is an independent marker of poor outcome and is linked to an immunosuppressive tumor microenvironment characterized by increased TAMs/CAFs and fewer cytotoxic lymphocytes (52). Furthermore, these metabolic perturbations intersect with the PI3K/AKT/mTOR pathway, a central node controlling cell growth and immunometabolism. Hyperactivation of PI3K/mTOR not only accelerates cancer cell proliferation and metabolic reprogramming but also skews immune responses, for instance by driving effector T-cell differentiation and function while fostering regulatory phenotypes that facilitate immune escape (53). Taken together, the evidence suggests that targeting these metabolic vulnerabilities, particularly those related to polyamine metabolism and glycolysis, may intersect with PI3K/mTOR signaling to influence tumor cell growth and relieve tumor-induced immunosuppression. Notably, our functional validation was limited to *in vitro* LUAD cell line models, and further studies using additional NSCLC models, patient-derived systems, or MPLC-specific experimental platforms, together with protein-level and phosphorylation-based validation, will be required to establish a causal mechanistic link.

However, several limitations should be acknowledged. First, the number of MPLC samples remains limited, and the presence of histological heterogeneity may introduce potential bias; thus, larger and more strictly annotated cohorts will be required for further validation. Second, although batch correction and integration analyses were performed, residual confounding from dataset origin, sample composition, and histological differences cannot be completely excluded. Third, the metabolic signatures in malignant cells were inferred from pathway enrichment analysis, future studies using Seahorse extracellular flux analysis, isotope-tracing metabolic flux assays, or mass spectrometry-based metabolomics will be required to confirm these metabolic dependencies. Lastly, the prognostic model was developed and tested primarily on public LUAD bulk RNA-seq datasets, and further validation in MPLC cohorts with matched transcriptomic and survival data is warranted. In addition, independent validation of MPLC-enriched Malignant_CAPS subpopulation using spatial transcriptomics or experimental approaches is currently constrained but will be an important direction for future studies.

Although this study is hypothesis-generating based on the premise that MPLC-associated malignant programs may contribute to immunosuppression and disease progression, our findings shed light on the cellular and metabolic heterogeneity in MPLC and underscore the importance of integrating metabolic-immune interactions into prognostic and therapeutic strategies in lung adenocarcinoma.

## 5 Conclusions

In this study, our integrative analyses of single-cell and bulk transcriptomic profiles delineate a previously unrecognized MPLC-enriched malignant subpopulation characterized by coordinated metabolic rewiring and immunoregulatory activity. By linking amino acid-driven metabolic reprogramming to MHC-II-mediated interactions with immunosuppressive CD4^+^ T cells and mast cells, our findings suggest a potential metabolism-immune interplay that may contributes to immune exclusion and poor prognosis in lung adenocarcinoma. The identification of key metabolic drivers, including SMOX and SMS, further connects polyamine metabolism to PI3K/mTOR-dependent tumor progression, highlighting actionable metabolic vulnerabilities. Together, these findings provide insight into metabolism-informed prognostic stratification and support the exploration of combined metabolic and immunotherapeutic strategies to improve clinical outcomes in lung adenocarcinoma.

## Data Availability

Publicly available datasets were analyzed in this study. This data can be found here: GSE131907, GSE200972, GSE31210, GSE30219, https://www.ncbi.nlm.nih.gov/geo/query/acc.cgi TCGA-LUAD, https://portal.gdc.cancer.gov.
