# Supplementary material for: Metabolic-immunoregulatory subtypes reveal prognostic and therapeutic insights in multiple primary lung cancer
Source: Front Immunol. 2026 Jul 15;17:1827965. doi: 10.3389/fimmu.2026.1827965 (PMC13415585; doi:10.3389/fimmu.2026.1827965)
Supplement: Supplementary file 1 [file DataSheet1.zip › Supplementary/Supplementary_Material_legends.docx]

Supplementary Material

# Supplementary Figures and Tables

## Supplementary Figures

**Supplementary Figure S1.** Single-cell transcriptomic landscape of all samples. (A) UMAP plot showing the distribution of cells from all samples, with each sample represented by a distinct color. (B) Quantitative evaluation of batch correction performance before and after Harmony integration. Local Inverse Simpson’s Index (LISI) and Silhouette score analysis showing dataset-level and cell-type-level mixing. (C) Correlation matrix and clustering of transcriptional profiles among different cell types. (D) Violin plots displaying the expression levels of canonical marker genes across identified cell types. (E) Heatmap showing the expression patterns of representative marker genes across all cells, highlighting major cell populations.

**Supplementary Figure S2.** Characterization of malignant epithelial cells and subtype-specific features. (A) UMAP plot showing the distribution of epithelial cells across different samples. (B) Stacked bar plot depicting the proportions of malignant and non-malignant epithelial cells within each sample. (C) Stacked bar plot comparing the proportions of malignant and non-malignant epithelial cells between the SPLC and MPLC groups. (D) Boxplot comparing the proportion of Malignant_CAPS cells among malignant epithelial cells between SPLC and MPLC samples, with statistical significance assessed by Wilcoxon rank-sum test. (E) Single-cell metabolic activity analysis showing relative pathway activity across malignant epithelial subpopulations, highlighting increased arginine and proline metabolism and histidine metabolism in the Malignant_CAPS subpopulation. (F) Bubble plot showing the top enriched GO biological processes in the Malignant_CAPS subpopulation.

**Supplementary Figure S3.** Myeloid cell diversity and MHC-II mediated interactions in SPLC and MPLC. (A) UMAP plot showing the clustering of myeloid cells into 21 distinct subsets. (B) Distribution of myeloid clusters across SPLC and MPLC samples, annotated by sample group. (C) Violin plots showing the expression of representative marker genes in each myeloid cell subpopulation. (D) The interaction strength via the MHC-II signaling pathway between MPLC-specific Malignant_CAPS, CD4⁺ T cell subpopulations, and mast cells within the SPLC and MPLC groups, respectively.

**Supplementary Figure S4.** Clinical relevance of prognostic genes derived from malignant subpopulations in LUAD. (A) Cross-validation curve of the LASSO Cox regression model showing the partial likelihood deviance across different values of log(λ). (B) Coefficient profiles of candidate prognostic genes generated by LASSO Cox regression. (C) Forest plot showing the multivariate Cox regression analysis of the metabolism-based risk score along with clinical factors in the TCGA-LUAD cohort. (D) Differential expression of model genes between tumor and adjacent normal tissues in TCGA-LUAD, assessed using Wilcoxon rank-sum test. (E) Protein-protein interaction network of model genes constructed via the STRING database. (F) KEGG pathway enrichment analysis of model genes based on STRING annotations.

## Supplementary Tables

**Supplementary Table S1**. Clinical information of lung cancer sample and single-cell RNA sequencing data.

**Supplementary Table S2**. The forward and reverse primer sequences for each gene analyzed in the RT-qPCR assays.
