# Supplementary figures and images for: Metabolic-immunoregulatory subtypes reveal prognostic and therapeutic insights in multiple primary lung cancer
Source: Front Immunol. 2026 Jul 15;17:1827965. doi: 10.3389/fimmu.2026.1827965 (PMC13415585; doi:10.3389/fimmu.2026.1827965)

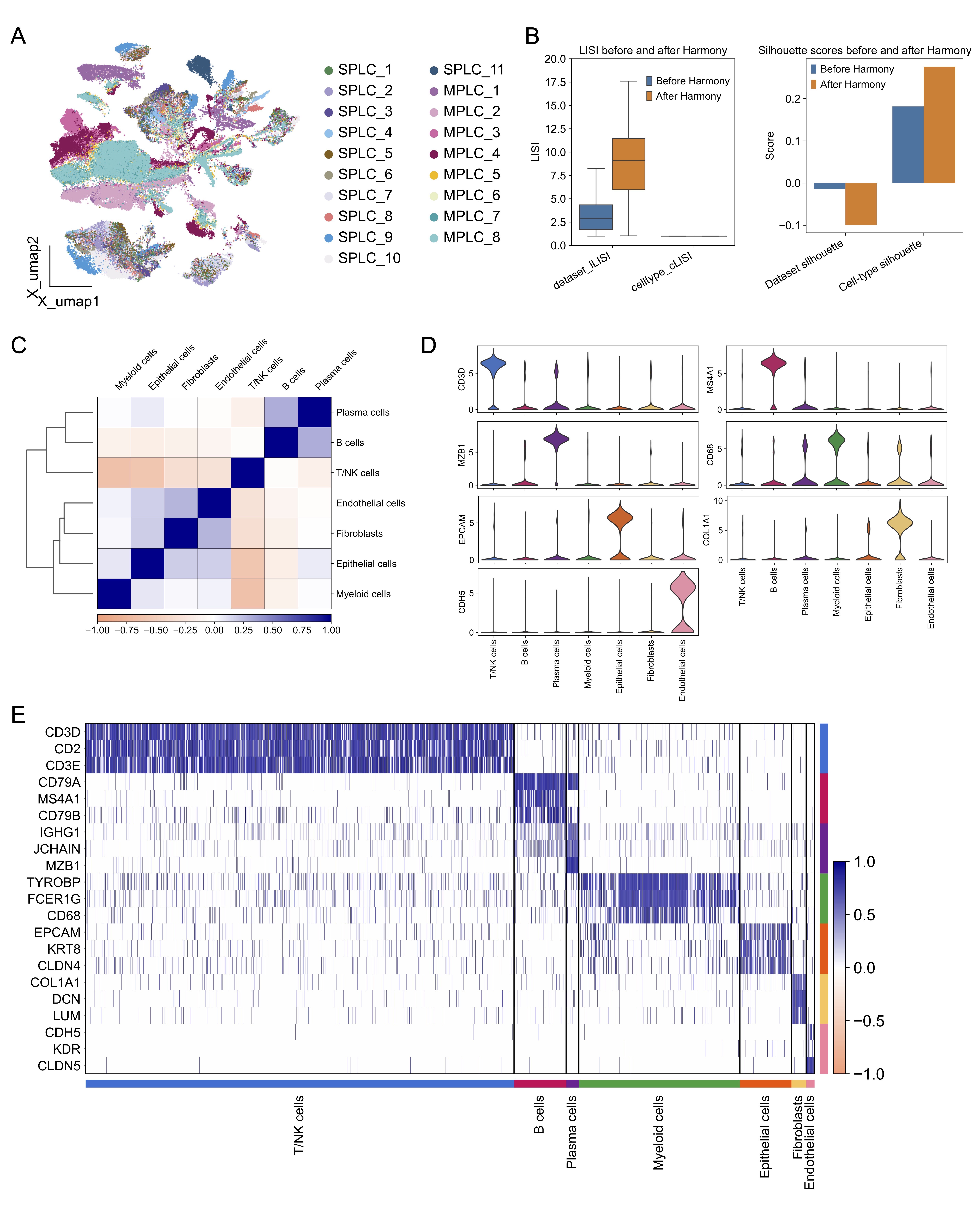

Supplement: Supplementary file 1 [file DataSheet1.zip › Supplementary/Figure S1.jpg]

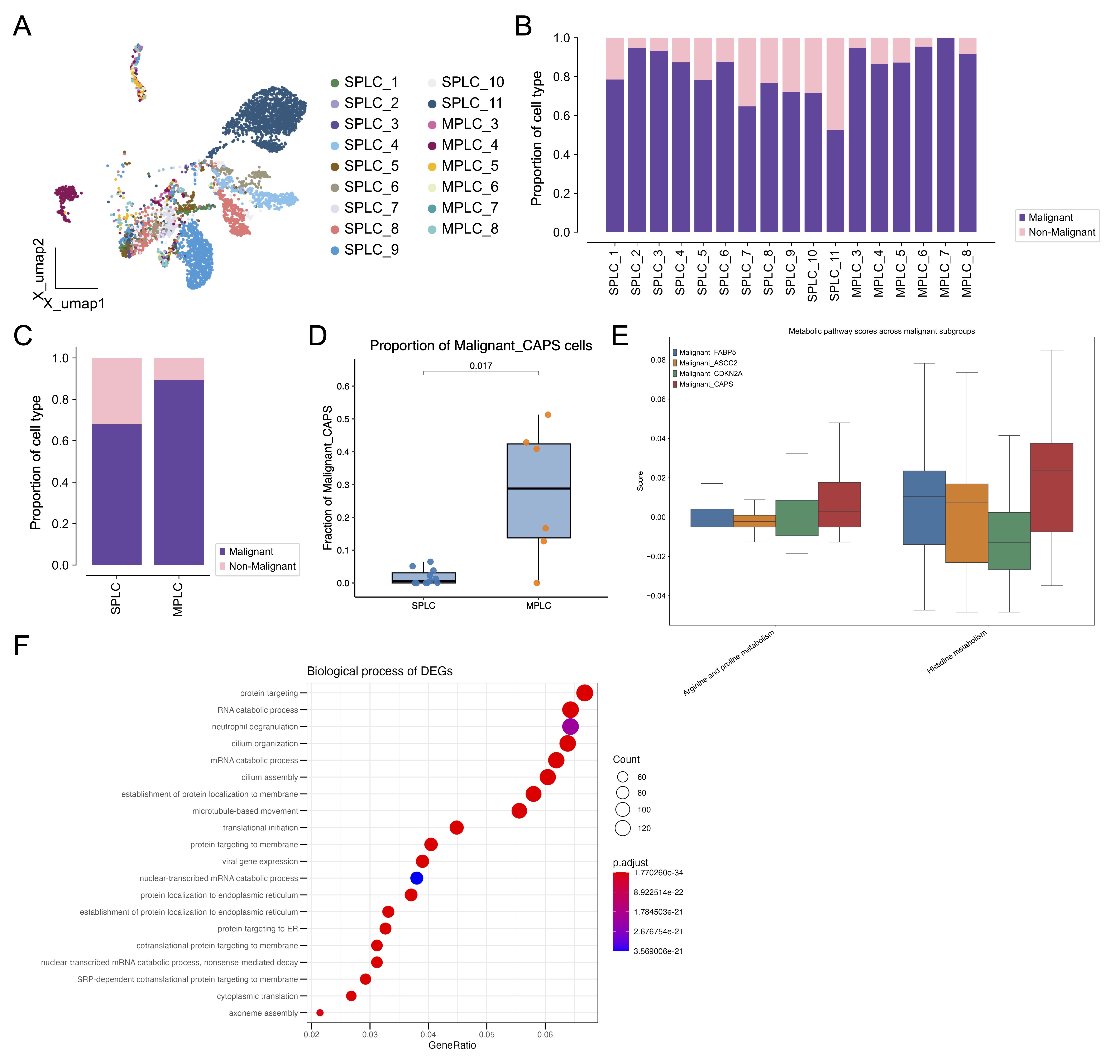

Supplement: Supplementary file 1 [file DataSheet1.zip › Supplementary/Figure S2.jpg]

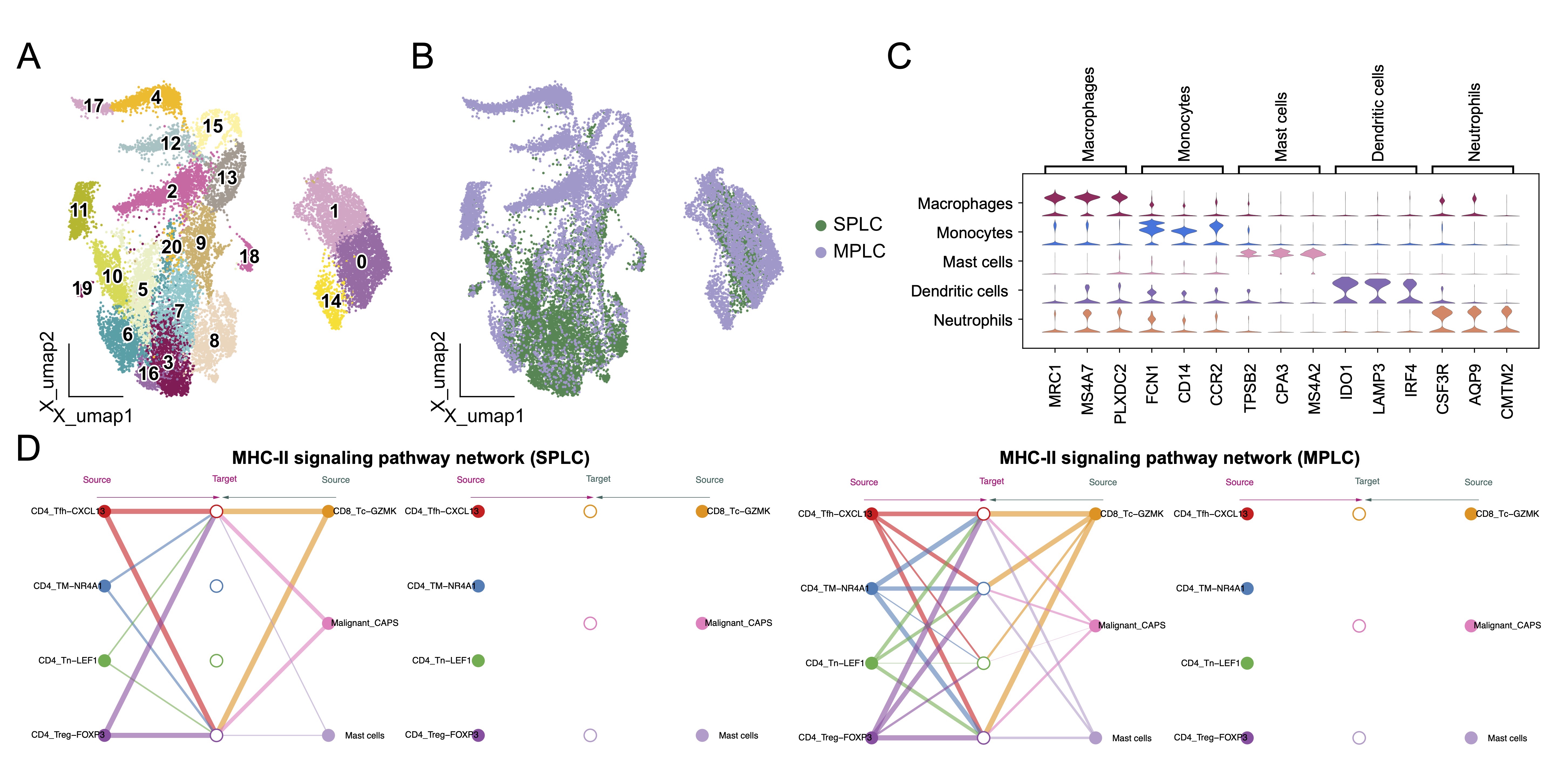

Supplement: Supplementary file 1 [file DataSheet1.zip › Supplementary/Figure S3.jpg]

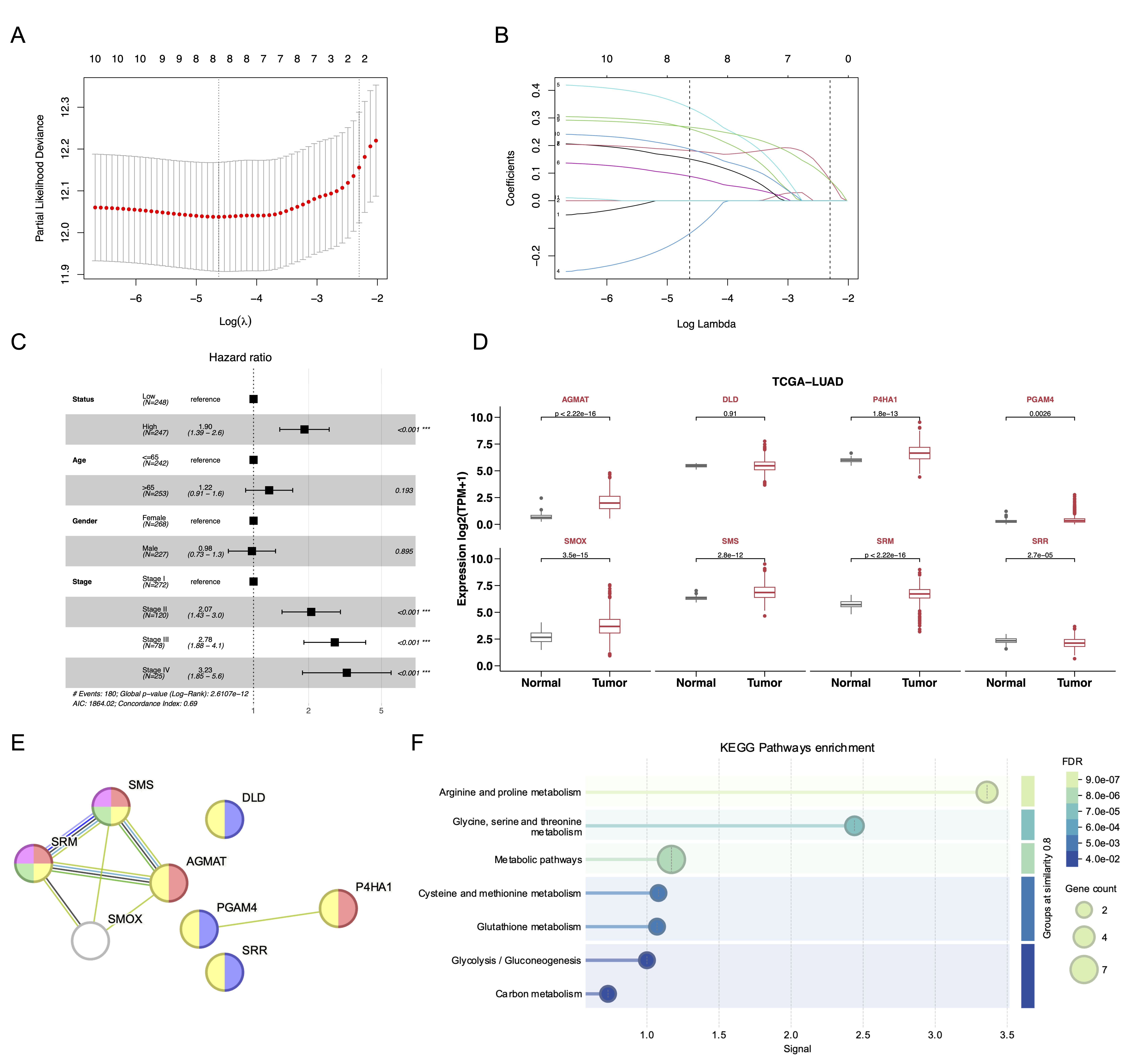

Supplement: Supplementary file 1 [file DataSheet1.zip › Supplementary/Figure S4.jpg]
